# Protective effects of hydrocolloid nanoemulsion coatings against myofibrillar protein oxidation in frozen trout

**DOI:** 10.3389/fnut.2025.1663357

**Published:** 2025-11-21

**Authors:** Ayşe Kara, Emre Çağlak, Barış Karslı

**Affiliations:** Department of Seafood Processing Technology, Faculty of Fisheries, Recep Tayyip Erdoğan University, Rize, Türkiye

**Keywords:** protein oxidation, aloe vera gel, hemp seed oil, nanoemulsion, *Oncorhynchus mykiss*

## Abstract

In this study, rainbow trout filets were coated using a dipping method with a nanoemulsion derived from aloe vera gel (AVG) and hemp seed oil (HSO; 5% HSO and 3% AVG). The filets coated with the nanoemulsion were then subjected to slow (−20 °C) and fast freezing (−40 °C) temperatures. The experimental groups were formed as follows: 20C (filets coated with pure water and frozen at −20 °C), 20N (filets coated with nanoemulsion and frozen at −20 °C), 40C (filets coated with pure water and frozen at −40 °C), and 40N (filets coated with nanoemulsion and frozen at −40 °C). Physicochemical analyses [pH, sulfhydryl groups (-SH), disulfide bonds (S-S), carbonyl content, myofibril fragmentation index, water-holding capacity, drip loss, TVB-N, and TBA] were performed monthly over a 6-month storage period. The results of this study indicated that none of the experimental groups exceeded the reported limit values for pH, TVB-N, and TBA during the 6-month storage period. It was observed that the filets coated with nanoemulsion and subjected to fast freezing (40N) delayed protein oxidation, as evidenced by the lower values of sulfhydryl groups, disulfide bonds, carbonyl content, and myofibril fragmentation index. Additionally, these filets maintained acceptable levels of quality parameters such as TVB-N, TBA, and pH, preserving their edibility. In conclusion, nanoemulsions containing AVG and HSO were found to improve the quality of fish products, helping them stay fresh for a longer period.

## 1 Introduction

In recent years, global food insecurity has been significantly influenced by factors such as the COVID-19 pandemic, armed conflicts, the climate crisis, and rapid population growth (1). Food insecurity is defined as limited access to safe, nutritious food in sufficient quantities and at reasonable prices. Inadequate access to such food, which is an essential component of living standards, can lead to an increased risk of obesity, diabetes, Alzheimer's disease, cardiovascular diseases, certain cancers, foodborne illnesses, and health-related conditions and deaths (2, 3). Within this context, the livestock sector plays a crucial role in providing high-quality protein to consumers. A significant portion of this need can be met through aquatic products, which offer valuable animal-based protein. While aquatic products are recognized for their high nutritional value and are widely consumed, they are highly susceptible to spoilage during various stages of production, processing, and distribution. Spoilage involves a series of reactions, such as lipid and protein oxidation, hydrolysis, and enzymatic and microbiological processes (4, 5). Fish muscle is a complex structure composed of sarcoplasmic, myofibrillar, and stromal proteins. Sarcoplasmic proteins (25%−30%) are soluble in low ionic strength solutions, play roles in the metabolic activities of the muscle, and contribute to the characteristic color of fish muscle. Myofibrillar proteins (MP; 70%−80%) constitute the primary structural components responsible for muscle contraction. Stromal proteins, on the other hand, include structural proteins such as collagen and elastin, and play a critical role in maintaining tissue integrity. The susceptibility of these protein fractions to free radicals directly affects both the quality attributes and technological processability of aquatic products (15). In this context, the propensity of muscle proteins toward oxidative processes particularly highlights the concept of protein oxidation. Protein oxidation is defined as covalent modification reactions occurring via reactive oxygen species (ROS; e.g., OH^·^, H_2_O_2_) or secondary products of oxidative stress and is considered an important quality indicator in meat and fish products (6). This process can occur either directly through the action of reactive oxygen species or indirectly via lipid peroxidation products. To prevent spoilage, various preservation techniques are employed including refrigeration, freezing, salting, canning, smoking, drying, and brining, all of which can extend the shelf life of aquatic products and help preserve their quality (7). Among these, freezing technology is one of the most commonly used traditional methods to preserve the near-fresh quality of aquatic products. However, freezing leads to the formation of ice crystals due to the high water content, and their size is influenced by the freezing rate. The size, shape, and distribution of ice crystals are closely linked to product quality. The formation of large ice crystals and their irregular distribution can cause irreversible damage to the cellular and tissue structures, leading to increased drip loss, dehydration, tissue softening, color deterioration, and enhanced lipid and protein oxidation after thawing. These factors contribute to a reduction in product quality and shelf life (8–11). Structures containing both hydrocolloids and lipids form a homogeneous matrix that protects aquatic products from physical and chemical stresses during freezing and storage. This matrix enhances water-holding capacity, preserves tissue integrity, and reduces lipid and protein oxidation. Nanotechnology improves the stability and functionality of these systems by controlling droplet size and distribution, ensuring uniform dispersion, optimizing interactions among components, and thereby maintaining the sensory, physical, and chemical quality of the product for a longer period. Specifically, nanoemulsions are widely used; these systems consist of two immiscible or partially miscible liquid phases (such as water and oil) dispersed in the form of droplets ranging from 20 to 200 nm (12). One of the key factors contributing to spoilage is oxidation. Fish, which contain a high levels of polyunsaturated fats and phospholipids, are particularly susceptible to oxidative spoilage. Lipid oxidation, quality defect characterized by the breakdown of lipids in the presence of prooxidant substances, results in the formation of oxidative products that cause rancidity. During lipid oxidation, certain sulfur-containing amino acids in the myofibrillar proteins of fish muscle have been shown to exhibit antioxidant effects, inhibiting lipid oxidation (13–16). However, in environments where lipid oxidation products accumulate excessively and antioxidant proteins are insufficient, protein oxidation can occur in fish muscle. Lipid free radicals formed as a result of rapid lipid oxidation in fish muscle can trigger protein oxidation, further accelerating the reaction. This results in simultaneous damage to both lipid and protein structures.

The effects of protein oxidation on aquatic product quality have not been elucidated as thoroughly as lipid oxidation in the literature. A deeper understanding of this mechanism is crucial for improving the sensory and nutritional quality of aquatic products and for the development of high-value-added products (17). The primary objective of this study was to investigate the effects of plant-based [aloe vera gel (AVG) and hemp seed oil (HSO)] nanoemulsions, on protein oxidation and meat quality changes in rainbow trout (*Oncorhynchus mykiss*) filets over a 6-month storage period. The nanoemulsions were prepared at different concentrations (1%, 2%, and 3% AVG, HSO/1:4 ratio). Characterization analyses were conducted, including pH, droplet size, polydispersity index, zeta potential, viscosity, turbidity, conductivity, color, whiteness index, transmission electron microscopy, and Fourier transform infrared spectroscopy, to identify the most suitable formulation, were conducted to identify the most suitable formulation. The selected nanoemulsion formulation was then applied to rainbow trout filets, which were subjected to both fast freezing (−40 °C) and slow freezing (−20 °C) conditions. The impact of the plant-based nanoemulsion coatings on protein oxidation and meat quality were evaluated throughout the 6-month period. Various analyses were performed to assess quality changes, including pH, sulfhydryl groups (-SH), disulfide bonds (S-S), carbonyl content, myofibrillar fragmentation index, water holding capacity, water activity, drip loss, texture, color, total volatile basic nitrogen (TVB-N), and thiobarbituric acid reactive substances (TBA). Based on the oxidation mechanism related to protein denaturation and degradation, the data obtained in this study were used to explore the impact of of coatings enriched with aloe vera gel and hemp seed oil nanoemulsions on the quality of trout filets subjected to both fast and slow freezing. The results were analyzed through multiple parameters to provide insights into their potential impact on product quality and shelf life.

## 2 Materials and methods

A total of 120 skinless rainbow trout (*Oncorhynchus mykiss*) filets, derived from 60 individual fish, were used as study groups. The fish were slaughtered on-site at the facility under hygienic conditions to minimize stress and ensure freshness, and filets were immediately processed and transported to the Fish Processing Technology Laboratory of the Faculty of Fisheries, Recep Tayyip Erdogan University. During transport, the filets were kept under temperature-controlled conditions (0–4 °C) to prevent microbial growth and oxidative deterioration, and the total transport time did not exceed 2 h, ensuring that the fish were still in the early post-mortem phase, immediately following the onset of rigor mortis, which is critical for maintaining meat quality and texture.

The aloe vera Barbadensis Miller (Stockton variety) plants were obtained in their pure gel form from a local producer in Antalya, Türkiye. 100% pure hemp (*Cannabis sativa* L.) seed oil, free from any additives or preservatives, was sourced from a commercial company (Tazemiz Ltd., Mersin, Türkiye). Both plant materials were handled under hygienic conditions and stored at appropriate temperatures prior to formulation of the nanoemulsions.

### 2.1 Preparation of nanoemulsion and their application on as coating material of rainbow trout (*O. mykiss*) filets

The nanoemulsion containing 5% hemp seed oil (HSO) and 3% aloe vera gel (AVG) was prepared based on the formulation identified as the most suitable formulation in our previous study (18, 19). The filets were coated on all surfaces by immersing them in this nanoemulsion solution using the immersion method. For the control group, the coating process was carried out with pure water. During the coating process, the filets were prepared under sterile conditions, the nanoemulsion was applied at 10% of the fish meat weight and the filets were immersed in the nanoemulsion for 5 min. Afterward, the filets were placed in a Laminar flow cabinet for 5 min to allow facilitate better penetration of the nanoemulsion (20).

### 2.2 Freezing of the filets and thawing prior to analysis

The coated and dried filets were subsequently packed in transparent sterile polyethylene bags. For the slow freezing process, the filets were frozen in a freezer (Vestel Eko NFY500, Turkey) at −20 °C, and the central temperature of the filets reached −18 °C after approximately 4 h. For the fast freezing process, an Alfa-laval freezer at −40 °C was used, and the central temperature of the filets reached −20 °C after approximately 2 h. The central temperature of the samples was continuously monitored during the freezing process using a wireless temperature probe. Once the central temperature of each rapidly frozen sample reached −20 °C, the samples were immediately transferred to a conventional freezer set at −20 °C. All slow and fast frozen samples were stored at −20 °C throughout the study period (6 months).

### 2.3 pH value analysis

The pH measurement was performed following the method described by Curran (21). In this method, the samples were diluted with distilled water in a 1:1 ratio, homogenized, and subsequently measured using a pH meter (Mettler Seven Compact–Toledo AG 8603).

### 2.4 Drip loss analysis

Drip loss (passive water loss) was determined by initially weighing the frozen filet samples. The samples were then suspended in polyethylene bags that were not exposed to any contact. After being stored at +4 °C for 24 h, the samples were gently blotted dry with paper towels and reweighed. Drip loss (%) was calculated as the difference between the pre- and post-suspension weights, divided by the pre-suspension weight, following the method described by Honikel (22).


(1) $$\begin{array}{l} \text{ Drip Loss }(\%)\text{ = }[(\text{Pre suspension weight }-\text{ Post }\\ \text{suspension weight})\text{/Pre suspension weight}]\;\times \text{ 100} \end{array}$$

### 2.5 Water holding capacity analysis

Five grams of the sample were weighed and centrifuged at 4,500 rpm for 30 min at 10 °C. After centrifugation, the supernatant was removed, and the pellet was weighed. The water holding capacity was then calculated using the following formula (23).


(2) $$\begin{array}{l} \text{Water Holding Capacity }(\%)\text{ = }[\text{1 }-\;(\text{Pellet weight/Initial }\\ \text{ weight of the sample})]\;\times \text{ 100} \end{array}$$

### 2.6 Determination of thiobarbituric acid (TBA) levels

The TBA analysis was conducted following the method outlined by Tarladgis et al. (24). In this procedure, 10 g of homogenized sample was weighed, and 97.5 ml of distilled water and 2.5 ml of 4N HCl were added. The mixture was then transferred to a boiling flask and subjected to distillation until 50 ml of distillate was collected. Following distillation, 5 ml of the distillate was taken, and 0.02 M thiobarbituric acid reagent was added. The mixture was incubated in a boiling water bath for 35 min. After cooling, the distillates were measured for optical density at 538 nm using a spectrophotometer. The resulting values were multiplied by a factor of 7.8 to determine the malondialdehyde (MDA) content in mg per 1,000 g of the sample.


$$\begin{array}{l} \text{TBA mg malondialdehyde}/\text{kg }=\text{ A}538\times 7.8 \end{array}$$

A538 = Absorbance value of the sample

### 2.7 Determination of total volatile basic nitrogen (TVB-N) levels

The determination of TVB-N in the study groups was performed according to the method described by Varlik et al. (25). In this procedure, 10 g of homogenized sample was placed in a distillation flask, to which 1 g of magnesium oxide (MgO), a few drops of silicone oil to prevent foaming, and 100 ml of distilled water were added. To collect the distillates and perform the titration, a 50 ml wide-necked Erlenmeyer flask was prepared by adding 10 ml of 3% boric acid (H_3_BO_3_), eight drops of Tashiro indicator, and approximately 100 ml of distilled water. The sample in the distillation flask was placed into the solution in the Erlenmeyer flask. The sample in the distillation flask was subjected to distillation for 15–20 min, and the collected distillate was titrated with 0.1 N HCl. The total TVB-N content was calculated using the following formula.


$$\begin{array}{l} \text{TVB}-\text{N }\left(\text{mg N}/100\text{ g}\right)\;=\text{ A }\times \;1.4\;\times \;100/\text{B} \end{array}$$

A: volume of 0.1 N HCl consumed (in ml)

B: sample weight

### 2.8 Myofibrillar protein extraction and determination of myofibril fragmentation index (MFI)

Myofibrillar protein preparation was carried out with slight modifications based on the methods described by Park and Xiong (26). A portion of minced fish muscle was homogenized with four isolation buffers containing 10 mM sodium phosphate, 0.1 M NaCl, 2 mM MgCl2, and 1 mM ethylene glycol tetraacetic acid (pH = 7.0). The homogenate was then centrifuged at 10,000 g for 15 min. The resulting pellet was washed again with 0.1 M NaCl to form the homogenate. After filtration, the supernatant was stored in different buffers for use in appropriate experiments. The MFI determination was carried out by measuring the absorbance value at 540 nm, after the pellet obtained from the samples homogenized in cold MFI buffer (40 ml of 0.02 M potassium phosphate buffer containing 100 mM KCl, 1 mM EGTA, 1 mM MgCl_2_, and 1 mM NaN_3_, pH 7.0) was centrifuged at 4 °C, (1,000 × g for 15 min) and resuspended. The MFI was then expressed by multiplying the measured absorbance by the dilution factor (200) (82).

### 2.9 Determination of carbonyl concentration

In this study, the carbonyl concentration for myofibrillar protein (MP) samples was determined using the 2,4-dinitrophenylhydrazine (DNPH) method. In this procedure, the MP suspension was mixed with a 10 mM DNPH solution and incubated at room temperature for 1 h. The mixture was then washed with 20% trichloroacetic acid for 5 min and centrifuged. After centrifugation, the resulting pellet was incubated with 3 ml of 6 M guanidine at 37 °C. Following the cooling process, the absorbance was measured at 370 nm and the carbonyl concentration was expressed as nM carbonyl per mg of protein (27).

### 2.10 Determination of sulfhydryl groups and disulfide bonds

The disulfide concentration of MP was determined using the 55′-dithiobis (2-nitrobenzoic acid; DTNB) reaction (28). The MP solution was mixed with 4.5 ml of 0.2 M Tris-HCl, 3 mM ethylenediaminetetraacetic acid, and 8 ml of 1% sodium dodecyl sulfate buffer, followed by the addition of 4 ml of this mixture to 0.5 ml of buffer B (10 mM Tris-HCl, 10 mM DTNB, pH 8.0). The resulting mixture was incubated at 40 °C for 25 min. At the end of the incubation, the absorbance of the samples was measured at 412 nm, and the disulfide concentration was determined.

### 2.11 Statistical analysis

The data obtained in this study were expressed as mean ± standard deviation (SD) based on 2–3 replicates per group (*n* = 2–3). Prior to statistical analysis, the data were tested for normality and homogeneity of variances to ensure the validity of parametric tests. Differences between groups with homogeneous variances were evaluated using a one-way ANOVA, with a significance level set at *p* < 0.05. All statistical analyses were conducted using the JMP 5.0.1 software package (SAS Institute Inc., NC, USA) (29).

## 3 Results and discussion

### 3.1 pH value

The changes in the pH values of the groups during the storage period are shown in Table 1. At the beginning of the present study, the pH value of fresh rainbow trout was measured at 6.21. During the 6-month storage period, statistically significant differences were observed in the pH values of both control and nanoemulsion-coated filets stored at −20 °C and −40 °C (*p* < 0.05). An increase in pH values was noted in both the control and nanoemulsion-coated groups throughout the storage period. The breakdown of protein structures due to microbial growth during storage leads to the formation of alkaline compounds, such as ammonia and amines, which contribute to the rise in pH of the product (30–32, 35). The pH value is an important freshness indicator for fish meat, typically to ranging between 6.5 and 7.0 (33). In the control and nanoemulsion-coated groups, this value was found to be 6.40 (at −40 °C) and 6.54 (at −20 °C) in the last month of storage. Based on the obtained data, none of the groups exceeded the specified freshness criteria. Additionally, the pH values of the nanoemulsion-coated filets were lower than those of the control groups. It is believed that the nanoemulsions containing hemp seed oil and aloe vera gel inhibited microbial growth, thus delaying the formation of volatile compounds. These findings align with previous literature. Yazgan (20) stated that nanoemulsion application resulted in a lower pH in fish meat. Yuvka (34) studied the changes in the pH value of rainbow trout filets stored in ice and treated with nanoemusions containing rosemary and oregano essential oils. Similarly to our study, Yuvka's (34) results showed that the pH gradually increased in the control group during storage, while fluctuations in pH were observed in the nanoemulsion-treated groups. A study focused on preserving the quality of aquatic products found that chitosan-based cinnamon-perilla essential oil, anthocyanidin, and collagen-enriched nanoemulsion films effectively extended the shelf life of aquatic products (35). It was also reported that pH values consistently increased during storage, with statistically significant differences observed between the nanoemulsion-treated groups and the control group.

**Table 1:** Changes in pH value in working groups during storage.

**Months**	**Groups**			
	**20C**	**20N**	**40C**	**40N**
Fresh	6.21 ± 0.01E^a^	6.21 ± 0.01D^a^	6.21 ± 0.01C^a^	6.21 ± 0.01C^a^
1	6.26 ± 0.02DE^a^	6.23 ± 0.01CD^ab^	6.24 ± 0.01C^ab^	6.20 ± 0.02C^b^
2	6.29 ± 0.02CD^a^	6.22 ± 0.01CD^ab^	6.21 ± 0.02C^b^	6.22 ± 0.03C^ab^
3	6.34 ± 0.01C^b^	6.28 ± 0.02CD^b^	6.45 ± 0.02A^a^	6.30 ± 0.01B^b^
4	6.45 ± 0.01B^a^	6.30 ± 0.02BC^b^	6.30 ± 0.02B^b^	6.26 ± 0.01BC^b^
5	6.47 ± 0.03B^a^	6.37 ± 0.01B^b^	6.32 ± 0.01B^bc^	6.30 ± 0.02B^c^
6	6.54 ± 0.01A^a^	6.49 ± 0.02A^ab^	6.42 ± 0.01A^b^	6.40 ± 0.01A^b^

The group coated with pure water frozen at −20 °C (20C), the group coated with nanoemulsion frozen at −20 °C (20N), the group coated with pure water frozen at −40 °C (40C), and the group coated with nanoemulsion frozen at −40 °C (40N). The data were calculated as mean (n = 3) ± standard deviation (SD). Different lowercase letters (a, b, c) in the same row indicate statistically significant differences between groups on the same storage day (p < 0.05). Different uppercase letters (A, B, C...) in the same column indicate statistically significant differences within the same group on different storage days (p < 0.05).

### 3.2 Drip loss

Changes in drip loss are presented in Table 2. Freezing is a commonly employed preservation method for perishable food products, particularly seafood. At temperatures below −10 °C, the growth of most spoilage and pathogenic microorganisms is effectively inhibited, making freezing a highly efficient method for extending shelf life and ensuring food safety. The results of this study indicated that samples frozen at −20 °C exhibited higher drip loss compared to those frozen at −40 °C. This is attributed to the rapid freezing process at −40 °C, which leads to the formation of smaller ice crystals, thereby minimizing tissue damage (36). A statistically significant difference in drip loss was observed both between groups and over storage months (*p* < 0.05). In the groups frozen at −20 °C, drip loss in the first month was 2.25% for the 20C group and 1.41% for the 20N group. By the final month, these values increased to 15.17% and 11.75%, respectively. For the rapid freezing method (−40 °C), first-month drip loss was 2.03% for the 40C group and 1.20% for the 40N group, rising to 9.09% and 7.31% by the final month. Previous studies supported these findings. Abbas et al. (37) reported that carp filets treated with essential oils (black seed, grape seed, and jojoba) and natural additives like chitosan exhibited lower drip loss compared to control filets during frozen storage. Similarly, nanoemulsions containing antioxidants derived from bamboo leaves, rosmarinic acid, and sodium lactate improved water-holding capacity in tuna, although a gradual decrease was observed during storage (38, 39). Sharifimehr et al. (40) developed edible coatings based on nanoemulsions containing varying concentrations of aloe vera gel powder and eugenol, finding that higher aloe vera concentrations significantly reduced drip loss in pink shrimp during refrigerated storage. In line with these findings, the reduction in drip loss observed in the nanoemulsion-treated groups compared to the control groups at both −20 and −40 °C in the present study indicates that hemp seed oil and aloe vera gel positively contributed to minimizing drip loss.

**Table 2:** Changes in drip loss values in working groups during storage.

**Months**	**Groups**			
	**20C**	**20N**	**40C**	**40N**
Fresh	^*^	^*^	^*^	^*^
1	2.25 ± 0.13D^a^	1.41 ± 0.08E^b^	2.03 ± 0.09F^a^	1.20 ± 0.38D^b^
2	6.33 ± 1.00C^a^	3.87 ± 0.08D^bc^	4.21 ± 0.06E^b^	2.83 ± 0.16C^c^
3	6.33 ± 1.00C^a^	3.88 ± 0.08D^b^	5.38 ± 0.33D^a^	3.50 ± 0.08C^b^
4	7.68 ± 0.05C^a^	6.04 ± 0.28C^b^	6.94 ± 0.55C^ab^	4.75 ± 0.35B^c^
5	12.30 ± 1.17B^a^	7.86 ± 0.25B^b^	8.06 ± 0.27B^b^	6.51 ± 0.46A^b^
6	15.17 ± 0.26A^a^	11.75 ± 1.46A^b^	9.09 ± 0.46A^c^	7.31 ± 0.13A^c^

The group coated with pure water frozen at −20 °C (20C), the group coated with nanoemulsion frozen at −20 °C (20N), the group coated with pure water frozen at −40 °C (40C), and the group coated with nanoemulsion frozen at −40 °C (40N). The data were calculated as mean (n = 3) ± standard deviation (SD). Different lowercase letters (a, b, c) in the same row indicate statistically significant differences between groups on the same storage day (p < 0.05). Different uppercase letters (A, B, C...) in the same column indicate statistically significant differences within the same group on different storage days (p < 0.05).

### 3.3 Water holding capacity (WHC)

The WHC is an important factor in determining the quality of frozen foods. A product with low WHC may result in undesirable quality issues, such as excessive water leakage and cooking loss. Decreases in WHC are known to be caused by the accumulation of cathepsins in the biochemical pathway, as well as the denaturation and aggregation of myofibrillar proteins (41). Increases in carbonyl content, which indicate protein denaturation, and decreases in sulfhydryl content can form protein cross-links. The cross-links affect the structure and spatial arrangement of myofibrillar proteins, ultimately reducing their WHC. It is well-established that physico-chemical properties of muscle foods—such as pH, cooking yield, WHC, and chemical composition (including moisture, protein, fat, and ash)—play a significant role in determining their overall quality and shelf life. Several studies have indicated that nanoemulsion treatment can help preserve cooking loss, fat binding, WHC, and texture properties in meat products (42–45). In the present study, the WHC value of fresh filets was found to be 61.52% (Table 3). During storage, the WHC values in the nanoemulsion-treated groups (20N and 40N) were significantly higher compared to the control groups (20C and 40C; *p* < 0.05). Regarding freezing rate, at the end of the storage period the WHC values of the slow-freezing groups (20C and 20N) were lower compared to the fast-freezing groups (40C and 40N; *p* < 0.05). This suggests that the slow freezing rate caused more structural deformation in the muscle tissue of the 20C and 20N groups, while the fast freezing rate resulted in less deformation.

**Table 3:** Changes in the water holding capacity (%) value in the working groups during storage.

**Months**	**Groups**			
	**20C**	**20N**	**40C**	**40N**
Fresh	61.52 ± 1.30A^a^	61.52 ± 1.30A^a^	61.52 ± 1.30A^a^	61.52 ± 1.30A^a^
1	22.80 ± 0.36B^b^	26.84 ± 4.75BC^ab^	28.73 ± 1.33B^ab^	31.02 ± 1.55BC^a^
2	19.97 ± 0.94BC^c^	28.84 ± 1.22B^b^	26.46 ± 0.06BC^b^	32.29 ± 2.09B^a^
3	20.57 ± 1.74BC^b^	21.90 ± 0.42CD^b^	23.60 ± 1.52CD^b^	27.88 ± 1.29CD^a^
4	21.19 ± 2.65BC^b^	19.61 ± 0.83D^b^	19.91 ± 1.34E^b^	27.24 ± 1.09CD^a^
5	18.31 ± 1.88C^b^	18.76 ± 1.45D^b^	19.04 ± 1.25E^b^	25.07 ± 2.25D^a^
6	18.79 ± 0.52BC^c^	19.47 ± 1.13D^bc^	21.16 ± 0.65DE^b^	24.36 ± 0.69D^a^

The group coated with pure water frozen at −20 °C (20C), the group coated with nanoemulsion frozen at −20 °C (20N), the group coated with pure water frozen at −40 °C (40C), and the group coated with nanoemulsion frozen at −40 °C (40N). The data were calculated as mean (n = 3) ± standard deviation (SD). Different lowercase letters (a, b, c) in the same row indicate statistically significant differences between groups on the same storage day (p < 0.05). Different uppercase letters (A, B, C...) in the same column indicate statistically significant differences within the same group on different storage days (p < 0.05).

### 3.6 Thiobarbituric acid (TBA) level analysis

**Figure 1:**
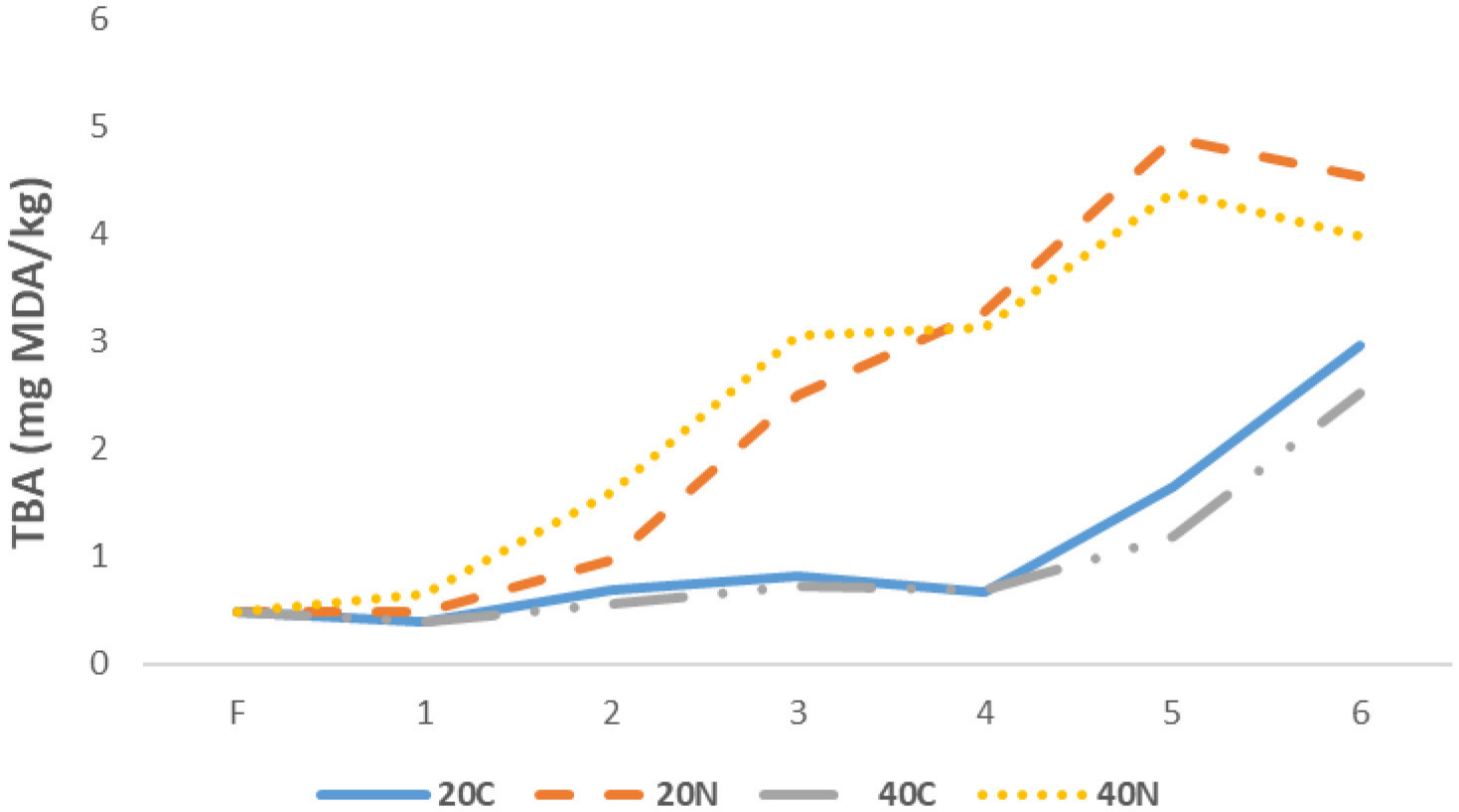
Changes in TBA values during storage among groups 20C: group coated with pure water frozen at −20 °C, 20N: group coated with nanoemulsion frozen at −20 °C, 40C: group coated with pure water frozen at −40 °C, 40N: group coated with nanoemulsion frozen at −40 °C, F: Fresh sample.

This study investigated the effects of nanoemulsion prepared with aloe vera gel and hemp seed oil, as well as freezing rate, on rainbow trout filet. The TBA values of all groups are presented in Figure 1. The TBA index is a widely accepted parameter for assessing lipid oxidation by measuring oxidation products, such as aldehydes, including malondialdehyde (MDA). During frozen storage, a significant increase in TBA values was observed across all samples (*p* < 0.05). This increase believed to be associated with the formation of ice crystals, an increase in prooxidants in the environment, and the promotion of oxidation reactions (46, 47). It has been reported that the acceptable maximum TBA level in fish, without adverse effect on safety and quality, is 5 mg MDA/kg (48). In the present study, the TBA levels of all groups were remained below this acceptable maximum level throughout the 6-month storage period. When the changes in the TBA values of all treatments were examined, it was observed that the values gradually increased toward the end of the storage period. However, the TBA values in the nanoemulsion-treated groups (20N and 40N) were significantly lower than those in the control groups (*p* < 0.05). The lower oxidation rates in the 20N and 40N groups treated with nanoemulsion enriched with aloe vera gel and hemp seed oil are believed to be due to the oxygen barrier and antioxidant properties of aloe vera gel and hemp seed oil. It is well-known that plant products rich in polyphenols exhibit scavenging activity by donating hydrogen atoms to free radicals, preventing the initiation of radical chains reactions, and inhibiting the formation of metal-catalyzed free radicals (49). Majidiyan et al. (50) reported that protein complex nanoparticles enriched with industrial hemp essential oil delayed the TBA values during storage of rainbow trout filets. In another study, Shokri et al. (51) showed that an edible active coating based on chitosan and *Ferulago angulata* (spiny caper) essential oil nanoemulsion significantly controlled the increase in lipid peroxidation in rainbow trout filets stored at 4 °C. In the present study, it was also found that filets subjected to rapid freezing (40C and 40N) exgibited lower TBA levels compared to those that were slowly frozen (20C and 20N).

### 3.7 Total volatile basic nitrogen (TVB-N) level analysis

**Figure 2:**
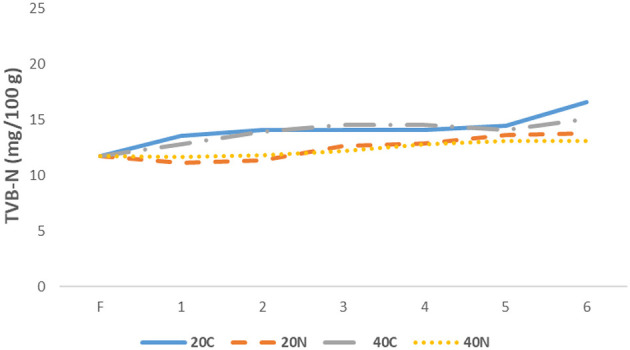
Changes in TVB-N level during storage among groups 20C: group coated with pure water frozen at −20 °C, 20N: group coated with nanoemulsion frozen at −20 °C, 40C: group coated with pure water frozen at −40 °C, 40N: group coated with nanoemulsion frozen at −40 °C, F: Fresh sample.

TVB-N analysis is widely recognized as a biomarker for protein hydrolysis-related spoilage in meat and fish products (52). An increase in TVB-N values is indicative of spoilage, primarily due to the heightened enzymatic and microbiological activities, which lead to elevated levels of ammonia, trimethylamine, dimethylamine, and other volatile nitrogenous compounds (53). It has been stated that the TVB-N values for fresh fish typicallyrange from 5 to 25 mg/100 g, with the upper threshold for acceptable TVB-N in consumable fish being 35 mg/100 g (54–56). In all experimental groups, TVB-N values exhibited a gradual increase during storage (*p* < 0.05). This study presented the changes in TVB-N values of rainbow trout filets treated with nanoemulsion during storage, as influenced by fast and slow freezing technologies, as shown in Figure 2. At the beginning of the storage period, the TVB-N value was 11.731 mg/100 g. During frozen storage, proteins, peptides, peptones, and amino acids undergo degradation into low molecular weight compounds, resulting in increase in TVB-N content across all groups. At the end of the storage period, TVB-N values were recorded as 16.61 mg/100 g for the control group at −20 °C (20C) and 15.08 mg/100 g for the control group at −40 °C (40C; *p* < 0.05). In the nanoemulsion-treated groups (20N and 40N), the final values were 13.83 mg/100 g and 13.14 mg/100 g, respectively. While an increase in TVB-N values was observed in both freezing conditions, the increase in the 40C group was lower compared to the 20C group. Furthermore, when compared to the control groups, the TVB-N values were significantly lower in the 20N and 40N groups, likely due to the effect of the nanoemulsion (*p* < 0.05). It is hypothesized that nanoemulsions, prepared using antimicrobial aloe vera gel and hemp seed oil, mitigated proteolysis and oxidative deamination of non-protein nitrogen compounds (which are linked to microbial activity), thereby reducing the production of volatile alkaline metabolites in the fish filets.

Numerous studies have investigated the use of antioxidant and antimicrobial nanoemulsions to regulate TVB-N levels, which are an indicator of fish quality (51, 57). Khedri and Roomiani (58) examined the chemical, microbial, and sensory properties of silver carp filets were found that increasing the concentration of nanoemulsions containing *Zataria multiflora* essential oil reduced TVB-N levels in the samples. Similarly, Durmuş (59) reported that the inclusion of olive oil nanoemulsions in rainbow trout filets showed the increase in TVB-N values. Wu et al. (43, 44) also stated that citrus oil nanoemulsions suppressed the changes in pH and TVB-N values of fish (*P. argenteus*) muscle during refrigerated storage, thus extending their shelf life. The findings suggest that the inhibitory effects of nanoemulsions on microbial growth and the delay in the formation of nitrogenous compounds likely contributed to observed reduction in TVB-N levels. These results are consistent with those reported in various studies in the literature (60, 61).

### 3.8 Determination of myofibrillar fragmentation index (MFI)

The MFI is an indicator of the protein denaturation and the myofibrillar protein (MP) degradation, which are key factors in determining meat tenderness. The MFI value indicates the degree of the structural deformation of myofibrils in muscle tissue (18, 19). According to the literature, during frozen storage, both the formation of ice crystals and oxidative reactions of lipids and proteins contribute to tissue damage, resulting in myofibrils breakdown and, consequently, an increase in MFI values (62–64). For example, a group of researchers investigated the effects of a fish gelatin-rosemary essential oil-based natural edible coating for the preservation of blunt snout bream (*Megalobrama amblycephala*) filets. They reported that the MFI values, which indicate protein and lipid oxidation leading to spoilage, were lower in the coated group (CT) compared to the control group (CK) (65). In the current study, the MFI value of fresh trout filets was determined to be 26.81. An increase in MFI was observed in all groups during the storage period. However, this increase was higher in the slow-frozen filet groups (20C and 20N) compared to the fast-frozen filets, with MFI values in the last month of storage being 159.37 for 20C and 125.70 for 20N (Table 4). This increase is believed to result from the larger and irregular ice crystals formed during the slow freezing process, which continue to grow during frozen storage, causing damage and significant fragmentation of muscle fibers. It is known that myofibrils gradually denature during storage, and that lowering the freezing temperature significantly reduces denaturation (66). The present study also suggested that the freezing rate has an effect on the deformation of myofibrils. Furthermore, it has been reported that the increase in MFI values is associated with breakdown of myonectin and accompanying actin in the I band of the MP sarcomere. The degradation of MP lead to the disruption of the structural integrity near the Z-line, which connects the sarcomeres within the myofibril (83). In the present study, MFI values were found to be lower in the nanoemulsion-treated groups (20N and 40N) compared to the control groups (20C and 40C), with nanoemulsion treatment showed a clearly positive effect on the myofibril integrity (*p* < 0.05).

**Table 4:** Changes in myofibril fragmentation index values in study groups during storage.

**Months**	**Groups**			
	**20C**	**20N**	**40C**	**40N**
Fresh	26.81 ± 0.51G^a^	26.81 ± 0.51F^a^	26.81 ± 0.51G^a^	26.81 ± 0.51F^a^
1	41.63 ± 2.06F^a^	27.80 ± 2.31F^c^	34.73 ± 0.50F^b^	26.91 ± 0.86F^c^
2	52.90 ± 0.78E^a^	43.03 ± 2.46E^b^	42.83 ± 2.42E^b^	35.23 ± 2.71E^c^
3	64.53 ± 1.53D^a^	55.37 ± 2.12D^b^	50.73 ± 0.64D^c^	47.63 ± 0.85D^c^
4	104.70 ± 0.46C^a^	65.27 ± 2.02C^c^	89.20 ± 1.78C^b^	56.17 ± 0.95C^d^
5	138.20 ± 1.57B^a^	84.73 ± 2.42B^c^	112.63 ± 2.28B^b^	75.50 ± 1.54B^d^
6	159.37 ± 1.36A^a^	125.70 ± 1.57A^c^	141.80 ± 1.05A^b^	107.33 ± 2.58A^d^

The group coated with pure water frozen at −20 °C (20C), the group coated with nanoemulsion frozen at −20 °C (20N), the group coated with pure water frozen at −40 °C (40C), and the group coated with nanoemulsion frozen at −40 °C (40N). The data were calculated as mean (n = 3) ± standard deviation (SD). Different lowercase letters (a, b, c) in the same row indicate statistically significant differences between groups on the same storage day (p < 0.05). Different uppercase letters (A, B, C...) in the same column indicate statistically significant differences within the same group on different storage days (p < 0.05).

### 3.9 Determination of carbonyl concentration

Carbonyl compounds are known as products of protein oxidation. Protein oxidation leads to protein cross-linking and aggregation of proteins, resulting in an increase in carbonyl and disulfide bonds, a decrease in active sulfhydryl groups, and a loss of protein functionality, including decreased solubility. These structural changes affect the seafood products, influencing factors as water-holding capacity of muscles (10, 67). During protein oxidation, amino acid side chain groups undergo modification, forming carbonyl derivatives. As a result, carbonyl compounds and free amines serve are important markers indicating the level of protein oxidation (68). The detection of carbonyl groups is one of the most common methods for detecting and measuring protein oxidation (69, 70). In the present study, the carbonyl concentration in fresh trout filets was initially determined to be 2.13 mmol/mg (Table 5). While fluctuations were observed within the groups during the storage period, the carbonyl concentration gradually increased in all groups. By the end of the storage period (6th month), the carbonyl concentration was measured to be 3.86, 3.38, 3.30, and 3.02 mmol/mg for the 20C, 20N, 40C, and 40N groups, respectively (*p* < 0.05). The highest carbonyl concentration was observed in the slow freezing groups, 20C and 20N. Frozen preservation was widely regarded the most effective methods for maintaining food quality criteria, including flavor, color, and nutritional value. However, the formation of ice crystals between cells during freezing can affect the quality of frozen products, with the extent of this impact depending on the freezing rate. In fast freezing, smaller and distributed ice crystals cause less damage to cell tissues and protein structure. Conversely, slow freezing results in the formation of larger and more irregular ice crystals, which can cause greater damage to food quality (71–75). This phenomenon was clearly evident in the 20C and 20N groups subjected to slow freezing in this study. In the fast-frozen groups, although the concentration was lower, the nanoemulsion-treated groups exhibited less damage to food quality, as evidenced by the reduced ice crystal formation, compared to the control groups. This suggested a positive effect of the nanoemulsion treatment (Table 5). Oxidative damage to lipids and proteins was also observed in the treatment groups, with a significant increase in protein carbonylation at slow freezing temperatures. Although lipid oxidative products can guide protein oxidation degradation, lipid oxidation and protein oxidation can occur independently or simultaneously. During protein oxidation, carbonyl compounds are formed, and certain amino acid side chains, such as lysine, histidine, proline, and arginine, play an crucial role in the carbonylation process (76, 77). In our study, a similar increase in carbonyl concentration was observed in relation to temperature, consistent with the findings in the literature.

**Table 5:** Changes in carbonyl concentration value (mmol/mg) in working groups during storage.

**Months**	**Groups**			
	**20C**	**20N**	**40C**	**40N**
Fresh	2.13 ± 0.06F^a^	2.13 ± 0.06D^a^	2.13 ± 0.06D^a^	2.13 ± 0.06C^a^
1	2.41 ± 0.01E^ab^	2.11 ± 0.24D^b^	2.49 ± 0.01C^a^	2.47 ± 0.06BC^a^
2	2.71 ± 0.08D^a^	2.45 ± 0.06CD^b^	2.41 ± 0.09C^b^	2.50 ± 0.10BC^ab^
3	3.02 ± 0.06C^a^	2.66 ± 0.09BC^ab^	2.77 ± 0.03B^ab^	2.54 ± 0.31BC^b^
4	3.12 ± 0.01C^a^	2.75 ± 0.01BC^b^	2.79 ± 0.06B^b^	2.50 ± 0.05BC^c^
5	3.43 ± 0.02B^a^	2.95 ± 0.02B^b^	2.84 ± 0.08B^b^	2.61 ± 0.15AB^c^
6	3.86 ± 0.19A^a^	3.38 ± 0.27A^b^	3.30 ± 0.11A^b^	3.02 ± 0.15A^b^

The group coated with pure water frozen at −20 °C (20C), the group coated with nanoemulsion frozen at −20 °C (20N), the group coated with pure water frozen at −40 °C (40C), and the group coated with nanoemulsion frozen at −40 °C (40N). The data were calculated as mean (n = 3) ± standard deviation (SD). Different lowercase letters (a, b, c) in the same row indicate statistically significant differences between groups on the same storage day (p < 0.05). Different uppercase letters (A, B, C...) in the same column indicate statistically significant differences within the same group on different storage days (p < 0.05).

### 3.10 Determination of sulfhydryl groups and disulfide bonds

**Figure 3:**
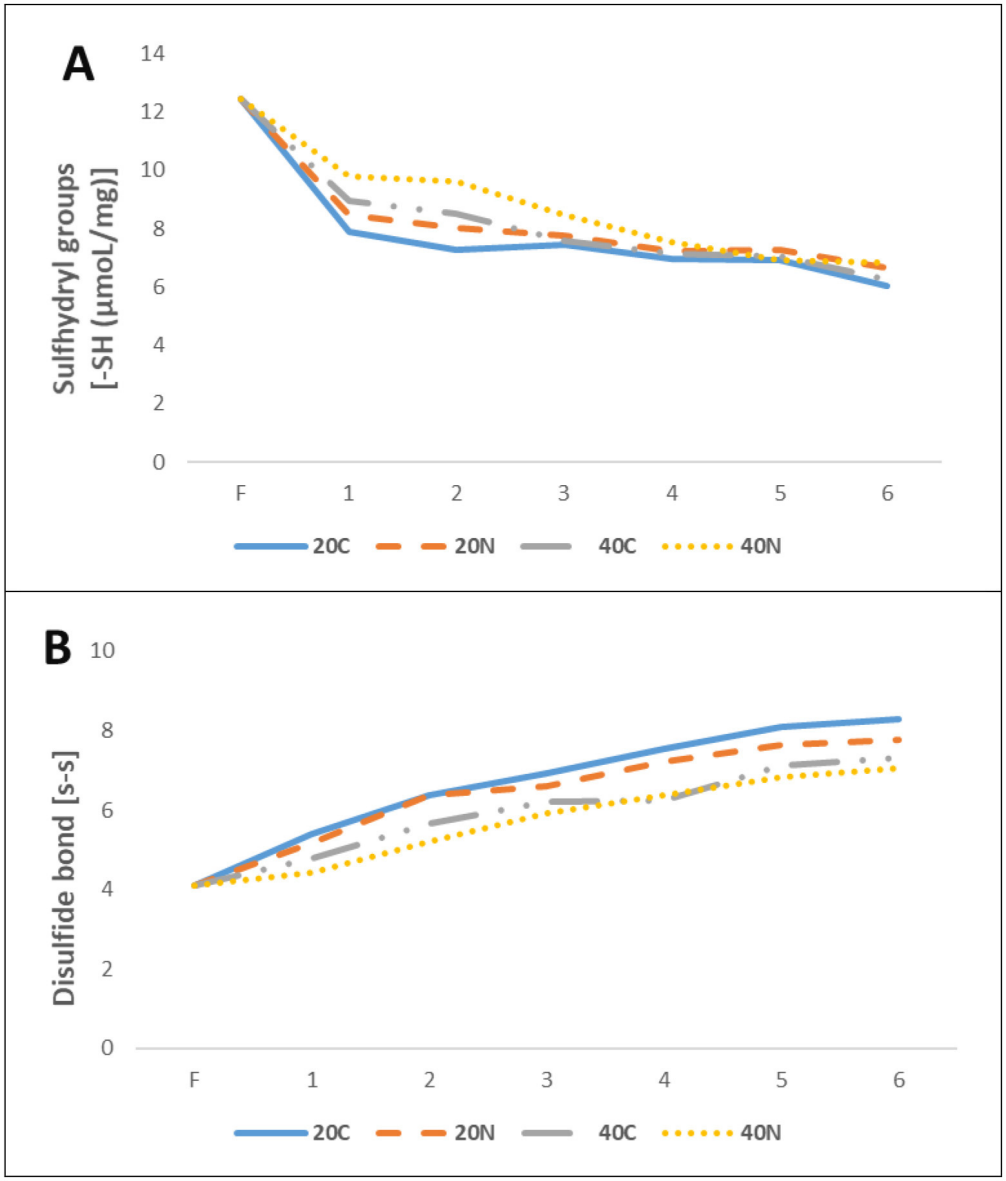
Changes in sulfhydryl group **(A)** and disulfide bond **(B)** values during storage among groups 20C: group coated with pure water frozen at −20 °C, 20N: group coated with nanoemulsion frozen at −20 °C, 40C: group coated with pure water frozen at −40 °C, 40N: group coated with nanoemulsion frozen at −40 °C, F: Fresh sample.

The loss of sulfhydryl groups is one of the most important indicators of protein degradation and increase in disulfide bonds associated with the loss of sulfhydryl groups reflects this change (56). During storage, a decrease in sulfhydryl groups was observed, accompanied by an increase in the number of disulfide bonds. In the present study, the sulfhydryl content of fresh trout filets was 12.45 μmoL/mg protein. After the first month of storage, sulfhydryl levels decreased to 7.89, 8.47, 8.96, and 9.78 μmoL/mg protein in the 20C, 20N, 40C, and 40N groups, respectively (Figure 3). A continuous decrease in sulfhydryl levels was observed from the first month of storage. In the last month of storage, the maximum decrease was 6.05 μmoL/mg in the 20C group, while the minimum decrease was 6.83 μmoL/mg in the 40N group (*p* < 0.05). In this study, the decrease in sulfhydryl content in MPs under slow freezing conditions, and in groups not treated with nanoemulsion, was interpreted as a result of structural changes in the protein due to the breakdown of myosin. This breakdown exposed and oxidized embedded sulfhydryl groups. Researchers have reported that sulfur-containing amino acids, such as sulfhydryl or cysteine residues, are particularly susceptible to oxidation by reactive oxygen species (78, 79). In this context, the relatively smaller decrease in sulfhydryl contents in the nanoemulsion-treated groups (20N and 40N) compared to the control groups (20C and 40C) suggests that the antioxidant properties of the aloe vera gel and hemp seed oil-based nanoemulsion helped reduce the levels of reactive oxygen species in the environment. This, in turn, helped to preserve the number of sulfhydryl groups. This finding was consistent with the results obtained from the TBA values.

In the present study, an increase in disulfide bonds was observed in parallel with the decrease in sulfhydryl groups across all groups. This was consistent with well-established understanding that sulfhydryl groups were oxidized to form disulfide bonds (80). Initially, the amount of disulfide bonds was 4.10 (fresh), and it gradually increased from the first month to the sixth month of storage. At the end of the storage, the disulfide bond levels were found to be 8.29 for the 20C group, 7.78 for the 20N group, 7.31 for the 40C group, and 7.05 for the 40N group (*p* < 0.05). During protein oxidation, concentration of sulfhydryl groups decreased, while the amount of disulfide increased. This study data suggested that sulfhydryl groups, exposed within the protein structure, were oxidized to form disulfide bonds. This oxidation process contributed observed decrease in sulfhydryl content and the corresponding increase in disulfide bonds during the final stage of storage (the 6th month). In the nanoemulsion-treated groups (20N and 40N), the lower levels of disulfide bonds were attributed to the antioxidant properties of the aloe vera gel and hemp seed oil in the nanoemulsion. These properties likely helped mitigate the oxidation process, thereby preserving the sulfhydryl groups. The findings of this study were consistent with those of Li et al. (81), who developed chitosan/clove essential oil nanoemulsion films to enhance oxidative stability and quality in refrigerated mirror carp filets.

## 4 Conclusion

This study investigated the effects of a plant-based nanoemulsion coating, containing aloe vera gel and hemp seed oil, on rainbow trout (*O. mykiss*) filets subjected to rapid and slow freezing conditions (−20 °C and −40 °C), with a focus on protein oxidation processes and changes in meat quality. Protein oxidation is a key factor contributing to quality deterioration in frozen fish, making the development of effective preservation strategies a critical challenge in the seafood industry. Natural antioxidant treatments, such as those provided by plant-based compounds, offer promising solutions to mitigate these quality losses. In this study, hemp seed oil and aloe vera gel were applied as natural antioxidants to enhance filet stability during frozen storage. The application of the nanoemulsion coating effectively preserved the physical, and chemical qualities of the filets compared to the control group. In particular, rapidly frozen filets (40 °C and 40N) treated with nanoemulsion showed the most pronounced improvements in pH, TVB-N, and TBA values, highlighting the combined effect of rapid freezing and natural antioxidant protection. Importantly, none of the groups exceeded the established quality limit values during the storage period. Evaluation of protein oxidation markers, including carbonyl groups, sulfhydryl groups, and disulfide bonds, further confirmed that rapid freezing combined with nanoemulsion treatment (40N) was the most effective approach for minimizing oxidative damage. These findings demonstrated that plant-based nanoemulsion coatings, when applied in combination with rapid freezing, could significantly reduce protein oxidation and preserve the overall quality of fish filets. This emphasizes the practical importance of such coatings as a natural, sustainable, and effective strategy to extend the shelf life of seafood products.

Furthermore, the study highlights opportunities for broader application and future research. Testing these nanoemulsions on other fish species and under diverse storage conditions could provide valuable insights into their versatility and help establish plant-based nanoemulsions as a standard approach in seafood preservation. Overall, this research contributes to advancing both scientific knowledge and practical strategies for improving the quality and safety of frozen aquatic foods.

## Data Availability

The datasets presented in this study can be found in online repositories. The names of the repository/repositories and accession number(s) can be found in the article/supplementary material.
